# Arabidopsis seedlings respond differentially to nutrient efficacy of three rock meals by regulating root architecture and endogenous auxin homeostasis

**DOI:** 10.1186/s12870-023-04612-1

**Published:** 2023-12-01

**Authors:** Tianjiao Zhang, Sainan Zhang, Shaohui Yang, Jianchao Zhang, Jiehua Wang, H. Henry Teng

**Affiliations:** 1 School of Environmental Science and Engineering, Tianjin University, Weijin Rd. 92, Nankai District, Tianjin, 300072 China; 2 School of Earth System Science, Institute of Surface-Earth System Science, Tianjin University, Weijin Rd. 92, Nankai District, Tianjin, 300072 China

**Keywords:** Basalt, Granite, Marlstone, Arabidopsis seedling roots, Auxin homeostasis

## Abstract

**Background:**

Plants show developmental plasticity with variations in environmental nutrients. Considering low-cost rock dust has been identified as a potential alternative to artificial fertilizers for more sustainable agriculture, the growth responses of Arabidopsis seedlings on three rock meals (basalt, granite, and marlstone) were examined for the different foraging behavior, biomass accumulation, and root architecture.

**Results:**

Compared to ½ MS medium, basalt and granite meal increased primary root length by 13% and 38%, respectively, but marlstone caused a 66% decrease, and they all drastically reduced initiation and elongation of lateral roots but lengthened root hairs. Simultaneous supply of organic nutrients and trace elements increased fresh weight due to the increased length of primary roots and root hairs. When nitrogen (N), phosphorus (P), and potassium (K) were supplied individually, N proved most effective in improving fresh weight of seedlings growing on basalt and granite, whereas K, followed by P, was most effective for those growing on marlstone. Unexpectedly, the addition of N to marlstone negatively affected seedling growth, which was associated with repressed auxin biosynthesis in roots.

**Conclusions:**

Our data indicate that plants can recognize and adapt to complex mineral deficiency by adjusting hormonal homeostasis to achieve environmental sensitivity and developmental plasticity, which provide a basis for ecologically sound and sustainable strategies to maximize the use of natural resources and reduce the production of artificial fertilizers.

**Supplementary Information:**

The online version contains supplementary material available at 10.1186/s12870-023-04612-1.

## Introduction

The increasing overexploitation of soil worldwide, especially in developing countries, is leading to a decline in the quantity and quality of soil and thus to soil degradation [1]. To reverse this trend and increase soil fertility, nutrients in soils must be replenished [2]. Meanwhile, mining operations generate about 7–17 billion tons of rock dust and byproducts from quarries annually [3], low-cost rock dust has been identified as a potential alternative to artificial fertilizers for more sustainable agriculture [4].

Improving soils with ground rocks is an ancient practice, as shown by limestone and gypsum for liming and apatite as phosphorus fertilizer. Powders of andesite and dacite are good substitutes for soluble fertilizers because of their high mineral dissolution rates [5], and some montmorillonite-rich volcanic ashes can increase the cation exchange capacity of poor soils. However, most silicate rocks have low nutrient release rates under laboratory conditions [6], and their composition of quartz, plagioclase, mica, and feldspar varies widely and generally contains large amounts of elements that are not essential for plant nutrition [7]. Therefore, although the release of elements by silicate weathering is the original source of mineral nutrients in soil, the direct use of rock meal in conventional agriculture is limited, and its agronomic efficacy and economic benefits have not been conclusively demonstrated [6].

When rock meal is used as a source of nutrients for plant growth and development, plants are not passive recipients but active participants in the process, either by dissolving minerals directly or by maintaining the diversity of weathering microorganisms in the rhizosphere. To complete their life cycle and produce yields, plants require 16 essential nutrients, including macro- and micronutrients [8]. Among them, nitrogen (N), phosphorus (P), boron (B), and sulfur (S) play fundamental roles as structural components; magnesium (Mg), zinc (Zn), iron (Fe), copper (Cu), molybdenum (Mo), and nickel (Ni) as cofactors for enzymes; potassium (K), sodium (Na), and chlorine (Cl) as electrolytes; and calcium (Ca) as messengers in signaling cascades. Plant nutrients, with the exception of nitrogen, are ultimately derived from the weathering of primary minerals. The dissolution of rock flour in the soil can be influenced by the composition of the soil solution and by plant actions, as well as by many factors such as climate, temperature, changes in pH in the rhizosphere, and redox and chelation by organic acids [8]. How plants perceive and adapt to the very low nutrient availability of rock flour and then trigger a series of internal signals to reprogram metabolic and genetic pathways to maximize mineral uptake and ensure optimal survival remains an open and fascinating question [9].

One of the most significant morphological adaptations in plants is changes in root system architecture [10] such as the spatial arrangement of roots at different ages and successions [11, 12], the proliferation of lateral roots [9], and the increase in the number and length of root hairs [13], which together increase the surface area available for nutrient uptake. This plasticity of the plant root system is determined by the intrinsic genetic program and is largely related to the interaction of plant hormones [14]. For example, roots can adapt to a nutrient-poor soil environment by constantly adjusting the endogenous distribution of auxin [15]. Inhibition of root growth by high nitrate supply in maize correlates with the decrease of auxin concentration in the root [16]. Cytokinins are also involved in controlling nutrient signaling and affect the homeostasis of a variety of nutrients, including N, P, S, and Fe, and are known to negatively regulate the response of Arabidopsis roots to inorganic phosphate (Pi) deficiency [17].

Deficiency of individual elements occurs much less frequently in natural ecological niches than under experimental conditions [6]. Although plant responses to combined stresses cannot be predicted simply on the basis of their responses to a single stress, there are few examples in the literature of changes in the architecture of the root system of plants exposed to complex nutrient limitation. In this work, we investigated how three finely ground rock powders can support the growth of Arabidopsis seedlings. We then investigated the rescue effects of trace elements and vitamins or individual macroelements (N, P, and K) when they were added back to the nutrient-poor medium. Considering the role of auxin in mediating plant responses to environmental stress, we also quantified endogenous auxin content and transcriptional changes of auxin biosynthetic genes under different supplementation conditions. By showing how plants sensitively and differentially adapt to heterogeneous geological conditions by regulating internal hormone homeostasis, this work helps us to develop rational strategies to improve the physiological use of mineral nutrients by plants and paves the way for recycling vast quantities of rock residues from the domestic mining industry for sustainable organic agriculture.

## Materials and methods

### Determination of mineral nutrient availability in rock powders

Three rock types, i.e., granite, basalt, and marlstone, were purchased from a mineral sorting company in Langfang, China. The rock powders were prepared from the massive rocks by crushing, grinding in a ball mill, and filtering through a 125 or 150 μm nylon mesh. The elemental composition of the rock powders was measured by mixed acid digestion (HF-HCl-HNO_3_-HClO_4_) and inductively coupled plasma (ICP) measurement (ME-MS61) according to a previously described method [18]. To measure extractable nitrogen (N), phosphorus (P), and potassium (K) in three rock meals, 400 g of granite, basalt, and marlstone were shaken separately in 1 L of a 1% sucrose solution (pH 5.7–5.8) at a speed of 30 rpm. The supernatant was collected after 0, 3, 7 and 15 days. Potassium persulfate digestion ultraviolet spectrophotometry was used to determine N content, ammonium molybdate spectrophotometry was used to determine P content, and ion chromatography was used to determine K content.

### Plant materials and growth conditions

Seeds of *Arabidopsis thaliana* ecotype Col-0 and the *DR5::GUS* reporter line in a Col-0 background was used for plant growth analysis. Arabidopsis seeds were sterilized with 75% ethanol for 5 min and 1.25% sodium hypochlorite (v/v) for 10 min before being vernalized at 4 ^◦^C in darkness for 3 days and sown on medium at 23 °C in a 16-h-light/8-h-dark cycle with a light intensity of 200 µmol m^−2^ s^−1^. To observe the effects of three types of rock mineral powder on the growth of Arabidopsis seedlings, in this work we chose to grow the seedlings in horizontally placed glass bottles rather than vertically placed plates because the mineral powder is much denser and sinks to the edge of the plate, resulting in an uneven texture of the medium and interfering with seedling growth. The medium contained 1% (w/v) sucrose, 0.65% (w/v) agar at pH 5.7–5.8. The positive control (CK) group contained half-strength Murashige and Skoog (½ MS) salt, the negative group contained no mineral (NA), and the experimental groups were NA medium supplemented with three rock meals (400 g/L basalt, granite, or marlstone). A lower agar concentration was used so that the roots could be easily removed from the medium to avoid damage to the roots. Only intact roots were counted and further stained for analysis. The micronutrients and organic nutrients (KI, H_3_BO_3_, MnSO_4_·4H_2_O, Na_2_MoO_4_·2H_2_O, CuSO_4_· 5H_2_O, inositol, glycine, VB1, VB6, and VB5) were added back to the experimental media according to their respective concentrations in 1/2 MS medium.

### Root morphological and histological analyses

Arabidopsis was grown for 15 days before morphological analysis. Photographs were taken of at least 30 plants per condition, and root length was measured using ImageJ software (https://imagej.net/). For GUS staining analyses, whole seedlings of Arabidopsis *DR5:: GUS* marker lines were collected after 7 days of growth for GUS staining and incubated at 37 °C for up to 18 h in staining solution (100 mM sodium phosphate buffer, pH 7.5, containing 10.0 mM EDTA, pH 8.0, 0.5 mMK_3_[Fe (CN)_6_] and 0.5 mM K_4_[Fe (CN)_6_], 0.1% Triton X-100 and 1.0 mM 5-bromochloro-3-indolyl-β-D-glucuronide). After staining, samples were washed with 70% (v/v) ethanol, prepared on microscopic slides, and observed using a Nikon microscope 50i equipped with a Nikon DS-Fi1C camera. At least 30 seedlings were processed and at least three independent experiments were performed, yielding the same statistically significant results.

### Endogenous IAA content measurements

To measure the endogenous concentrations of free IAA, 15-day-old seedlings were collected. According to previous methods [19], 300 mg samples were incubated overnight at -18 °C to precipitate the lipid material. After centrifugation at 12,000 rpm for 15 min at 4 °C, the precipitates and plant debris were removed and the supernatant was purified using a C18 Sep-Pack column (Waters Corporation). The eluent was then dried under a stream of nitrogen and dissolved in 80% methanol. IAA was analyzed quantitatively using UPLC-MS /MS (Acquity TQD, Waters, Manchester, United Kingdom). We pooled tissues from 30 seedlings for a biological sample, and each sample was technically tested three times.

### Gene expression analyses by qRT-PCR

Total RNA was extracted from 15-day-old Arabidopsis roots using the EasyPure Plant RNA Kit (TransGen Biotech, Beijing, China). One µg of total RNA was used to synthesize cDNA using the EasyScript FirstStrand cDNA Synthesis SuperMix (TransGen, China). The qRT-PCR analysis of cDNA was performed with the PikoReal96 Real-Time PC System (Thermo Fisher, Shanghai, China) using the Real Master Mix (SYBR Green) (Newbio, China). The thermal PCR cycling conditions were as follows: 95 °C for 2 min, followed by 44 cycles of 94 °C for 20 s and 59 °C for 20 s, 72 °C for 40 s. All primer sequences were designed using the Primer premier 5.0 program (http://www.premierbiosoft.com/primerdesign/) and are shown in Table S1. Relative transcript levels were analyzed using the ΔΔCT method (fold change = 2^−[ΔΔCT]^) as described previously [20]. All reactions were repeated three times with three independent combined samples, and the *AtEF1α* gene was used as an internal standard.

### Statistical analysis

All statistical analyses were performed using SPSS26.0 software (https://www.ibm.com/cn-zh/analytics/spss-statistics-software) and Microsoft Excel 2021. Student’s *t* test was used for single comparison and Duncan’s test for multiple comparison. Results were based on at least three replicates from three independent experiments.

## Results and discussions

### Compositions of three rock meals and their support for the overall growth of Arabidopsis seedlings

Many plant nutrients in rocks occur in small amounts or traces as accessory minerals, such as S in sulphides and P in apatite, and sometimes as substituents within the structures of rock-forming silicate minerals [6]. As shown in Table S2, basalt powder contains less K compared to the other two rock powders, marlstone powder contains much less Na, and granite powder is particularly poor in Fe, Mg, and Ca (Table S2). We then measured the amount of N, P, and K released from the three rock powders after they were slowly shaken in a sucrose solution for 3, 7 and 15 days, respectively. These extractable nutrients, which represent the availability of elements that can be used by plants for growth at day 7, were only 12.5% N, 2.9% P, and 6.6% K of those in the normal ½ MS medium (referred to as the CK group). Approximately, the marlstone extract contained twice as much dissolved N, while the basalt extract contained almost twice as much P and K as the other two rock powders (Table 1).

**Table 1:** Content of N, P and K elements in 1/2 MS medium and the amount of N, P and K (mg/L) released from the three rock meals after 3, 7 and 15 days of slow shaking in sucrose solution

1/2 MS		N	P	K
		490.00 ± 5.40	18.60 ± 0.30	390.00 ± 2.10
**Basalt**	Day 0	21.40 ± 0.31	0.31 ± 0.02	11.30 ± 0.11
	Day 3	25.10 ± 0.54	0.53 ± 0.03	22.70 ± 0.35
	Day 7	27.00 ± 0.67	0.54 ± 0.03	25.60 ± 0.53
	Day 15	29.12 ± 0.45	0.54 ± 0.08	26.15 ± 0.47
**Granite**	Day 0	25.10 ± 0.52	0.15 ± 0.01	4.93 ± 0.09
	Day 3	26.00 ± 0.51	0.17 ± 0.01	13.50 ± 0.25
	Day 7	37.30 ± 0.75	0.20 ± 0.02	15.20 ± 0.29
	Day 15	39.58 ± 0.48	0.21 ± 0.01	16.31 ± 0.23
**Marlstone**	Day 0	52.60 ± 1.02	0.14 ± 0.01	10.70 ± 0.12
	Day 3	58.20 ± 1.04	0.24 ± 0.02	15.60 ± 0.14
	Day 7	61.40 ± 1.13	0.28 ± 0.04	18.00 ± 0.34
	Day 15	62.79 ± 1.07	0.29 ± 0.03	19.17 ± 0.28

**Fig. 1:**
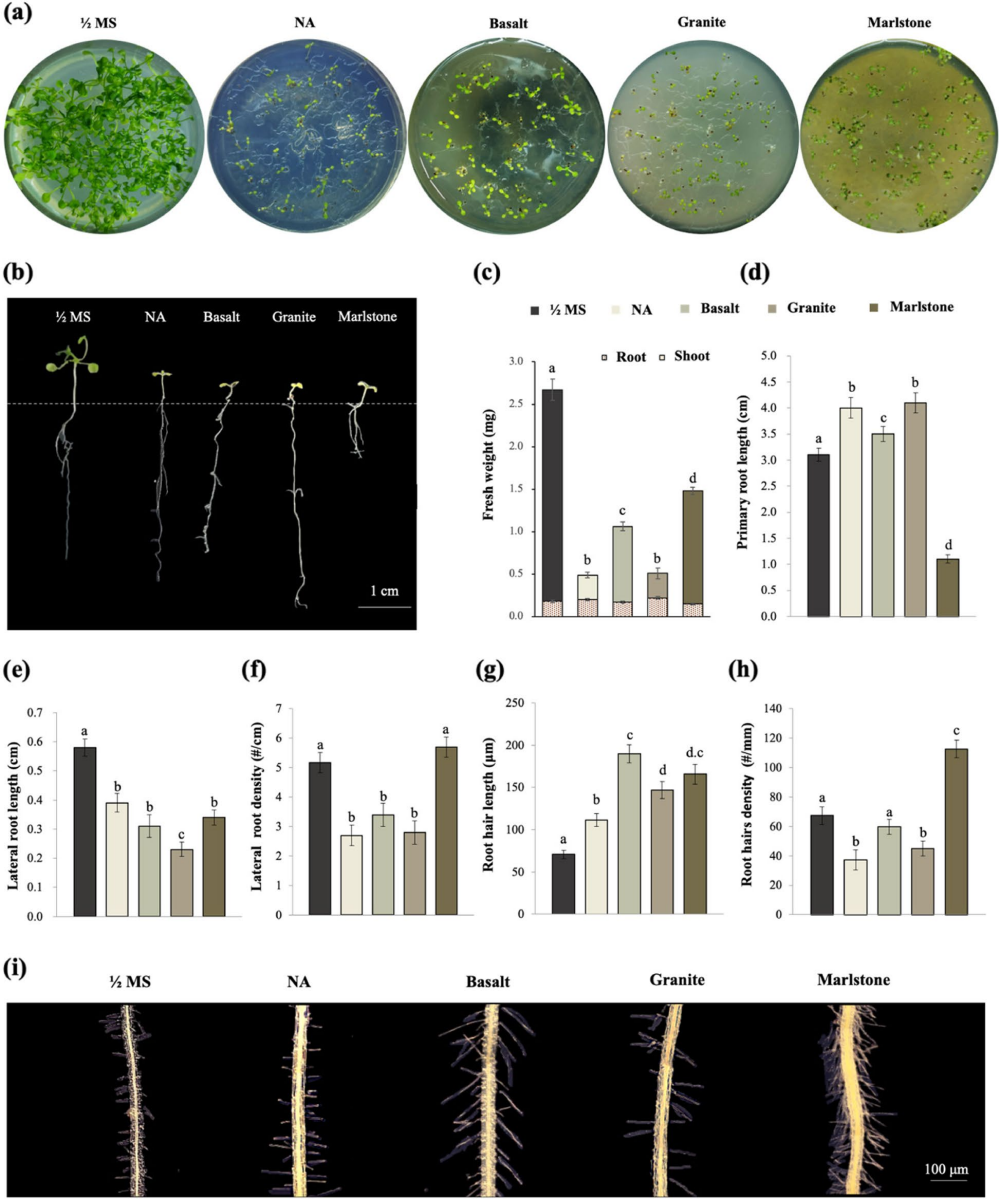
Growth indices of 2-week-old *Arabidopsis thaliana* seedlings growing on three rock meals. **a**-**b** photographs of Arabidopsis seedlings; **c** fresh weight; **d** primary root length; **e** lateral root length; **f** lateral root density; **g** root hair length; **h** root hair density; **i** photographs showing root hairs. Data are presented as mean (*n* = 30) ± SD. Different letters (**a**, **b**, **c**, and **d**) indicate significance with *p* < 0.05

Basaltic rocks contain various essential elements (Ca, Mg, K, P, S, and Fe) and beneficial elements (Na and Si) for crops [21], and have the highest cation release rates after phonolitic volcanic rocks [22]. Granite (silicate) rock dust is an insoluble byproduct of quarry operations. In general, the dissolution rates of felsic rocks (e.g., granite) are lower than those of mafic rocks (e.g., basalt). When used as a fertilizer in the wheat belt of southwestern Australia, it did not prove to be an effective fertilizer for wheat and clover crops because of its content of nutrient elements (1.9% K and 0.3% P and negligible N) [23]. However, in another study with low electrical conductivity and alkaline pH, the addition of granite powder significantly increased crop yield compared to the other amendments (up to 75% higher than in soil without amendment) [24]. Three rock meals had no effect on Arabidopsis seed germination when added at 40 g/100 mL to a nutrient-free agar medium. However, after 14 days, seedling growth was significantly and differently affected by the three rock meals (Fig. 1a-b). Compared with the roots of seedlings grown on normal ½ MS medium, the roots of seedlings grown on marlstone medium were much shorter but maintained normal geotropism, whereas the roots of seedlings grown on basalt or granite medium were much longer and distributed radially in the surface without obvious geotropism (Fig. S1). Compared with seedlings from the CK group, seedlings grown on medium without nutrients (referred to as the NA group) retained only 17% of the overall fresh weight, and the addition of basalt, granite, and marlstone increased this value to 41%, 18%, and 58%, respectively (Fig. 1c).

### Three mineral meals differentially affect the root growth of Arabidopsis seedlings

The root system of plants is the organ that exhibits the greatest morphological plasticity in response to various nutritional stresses. Highly branched, actively growing root systems are often associated with greater efficiency in nutrient uptake than those lacking such root characteristics. In this work, the primary root (PR) length of seedlings growing on a NA medium was 34% longer than that of CK seedlings, and the addition of basalt and granite increased PR length by 13% and 38%, respectively (Fig. 1b, d). In contrast, marlstone caused a significant 66% decrease in PR length (Fig. 1b, d). Rock powder of varying fineness prepared by filtration with 125 or 150 μm nylon mesh only slightly improved root phenotypes (Fig. S2). Compared to CK seedlings, the average length of lateral roots (LRs) of seedlings growing on NA medium was significantly shortened by 27%, and on the medium amended with basalt, granite, and marlstone, the reduction was 52%, 65%, and 40%, respectively (Fig. 1e). The density of LRs was 42%, 36%, and 40% lower on medium NA, basalt, and granite, respectively; than in the control group (Fig. 1f). However, the density of LRs was virtually unchanged on the marlstone medium compared to the CK group (Fig. 1f). The proliferation of LRs is known to be a response of many plant species to locally high nitrate or ammonium concentration and is a common adaptation phenomenon in the nutrient-rich zone [25]. Meanwhile, LRs have been reported to be suppressed in response to low nitrate supply [26] and high C/N ratio [27], which is likely the cause of the reduced LR length and density observed in this study. Doubling the amount of rock meal in the medium did not improve any of the above root phenotypes, with the exception of marlstone, where double addition increased primary root length by 20%, although this phenotypic improvement did not result in a corresponding increase in fresh weight (Fig. S3).

Another striking phenotypic trait observed in the current study was the changes in root hairs, whose average length increased significantly on all three rock meals (Fig. 1g-i). Root hair density increased in plants grown on marlstone but decreased in plants grown on granite and NA (Fig. 1h-i). As the most intimate interface between soil and root, root hairs increase the soil volume explored by roots, make soil pores more accessible [28], allow wider distribution of root exudates [29], and are involved in the formation of rhizosheaths [30]. Changes in root hair morphology are considered the cheapest way for a plant to increase root system surface area [13], although they are also associated with significant metabolic costs or increased susceptibility to pathogens [31]. Regarding P procurement, root hairs tend to be sparse in plants with adequate P content but increase in length and density as P content decreases [32]. It is believed that there is an optimal root hair length and root hair density for the most efficient P uptake, with the greatest efficiency achieved by increasing the length and longevity of root hairs rather than increasing their density [33].

In conclusion, Arabidopsis seedlings grown without nutrient supply but with only three types of rock meal showed different root growth characteristics and adaptation strategies to cope with the specific nutrient stress. In the case of basalt and granite, the seedlings developed longer primary roots and/or root hairs to forage on the surface of the medium (Fig. 1d and g), which basically resembled the characteristics of seedlings growing on the NA medium. But on the medium supplemented with marlstone, the seedlings instead developed shorter primary roots with denser root hairs (Fig. 1d and h) and showed almost normal geotropic growth for foraging inside the medium. This shows that even if the nutrient availability of the different rock meals is all poor, the plants can distinguish them and respond accordingly during root organogenesis and morphogenesis. Another phenomenon worth mentioning is that the number and density of lateral roots were reduced to varying degrees on all three media (Fig. 1e-f). Compared to the previous report that Pi-limiting conditions arrests primary root growth and promotes lateral root proliferation in Arabidopsis plants [34], our work revealed that during a large-scale shortage of mineral nutrients, plants could divert their limited resources from lateral root growth and development, and the adaptation strategy focused mainly on primary roots and root hairs.

### Effects of micronutrients and organic nutrition re-supplementation

**Fig. 2:**
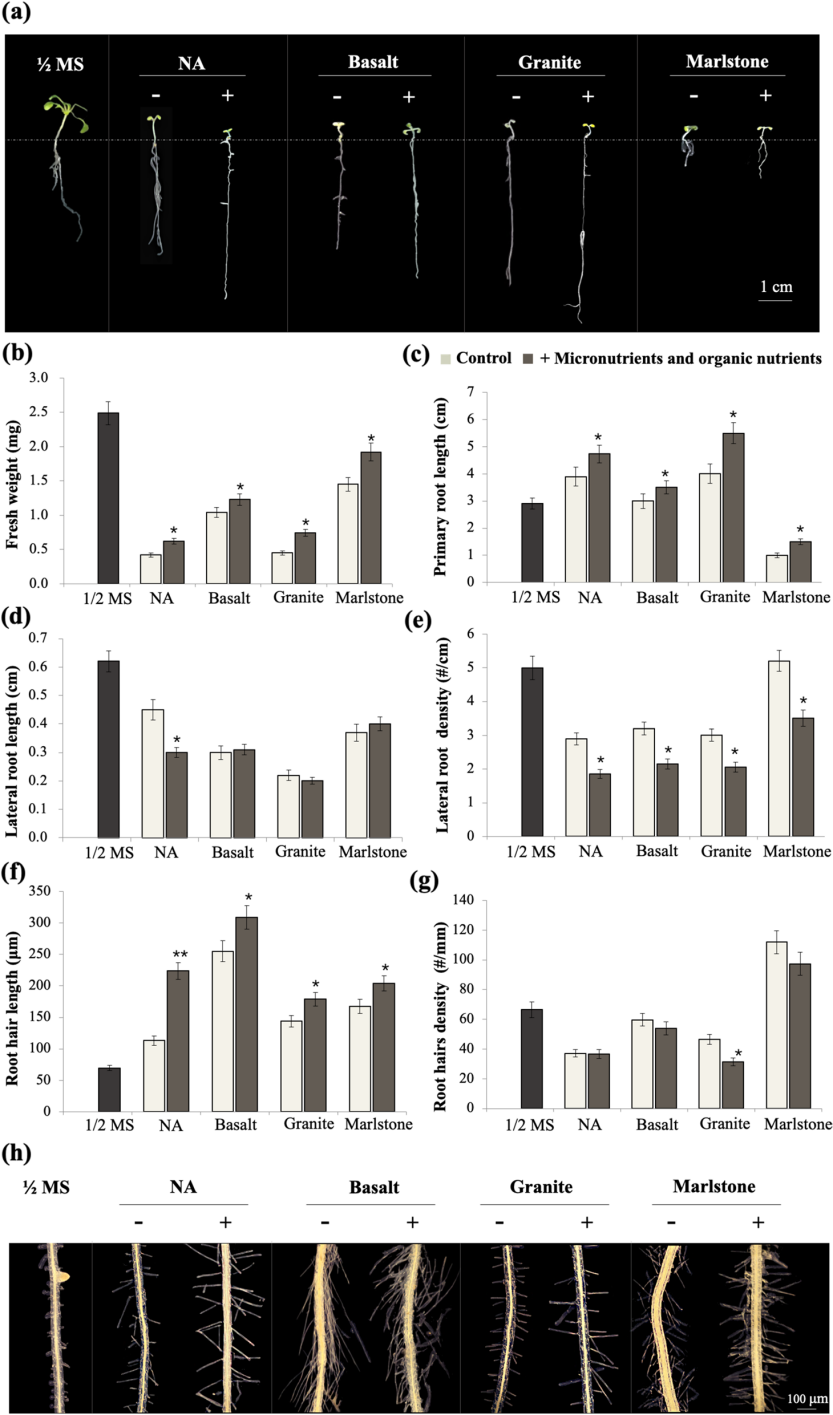
Effects of simultaneous addition of micronutrients and organic nutrients to growth media on 2-week-old Arabidopsis seedling growth. **a** photographs of Arabidopsis seedlings with or without added nutrients; **b** fresh weight; **c** primary root length; **d** lateral root length; **e** lateral root density; **f** root hair length; **g** root hair density; **h** photographs showing root hairs. Data are presented as mean (*n* = 30) ± SD. * and ** indicate *p* < 0.05 and *p* < 0.01, respectively

In this section, we simultaneously added micronutrients and organic nutrients to the growth medium according to their respective concentrations in ½ MS medium and then examined their restorative effects on the growth of Arabidopsis seedlings. First, the addition of these two classes of nutrients increased the fresh weight of seedlings fed on NA and three rock meals by 20–67% compared to their corresponding values without the addition (Fig. 2a-b). Second, the primary root length of the seedlings was increased by the addition of micronutrients and organic nutrients (Fig. 2a, c), meaning that the longer PRs of the seedlings growing on the NA, basalt, and granite were even longer (phenotypically enhanced), but the shorter primary root phenotype on marlstone was elongated (phenotypically attenuated).

The simultaneous addition of micronutrients and organic nutrients did not dramatically affect the average length of lateral roots, except that this index was further reduced on the medium NA (Fig. 2d), and the density of lateral roots was further reduced on all media (Fig. 2e). As for root hairs, the addition of micronutrients and organic nutrients increased root hair length by 21-26% without obviously affecting root hair density (Fig. 2f-h). Thus, the increase in fresh weight of Arabidopsis seedlings on rock meal after the simultaneous addition of organic nutrients and trace elements was probably due to the increase in the length of both PRs and root hairs.

### Effects of N, P, and K macronutrients re-supplementation

Among essential plant nutrients, N is the most limiting factor, P and K are the second and third most important nutrients needed for plant growth, metabolism, development and productivity. This explains why the fertilizer industry focuses almost exclusively on producing fertilizers containing these three macronutrients, rather than secondary and micronutrients [35]. When grown under limited P availability, roots exhibit inhibited PR elongation and the concomitantly increased LR formation [36, 37] to improve the ability to forage P from the usually P-enriched topsoil horizon [10, 38, 39]. In contrast to low P, reduced N availability stimulates PR and particularly LR elongation but not LR initiation [9, 40] with an almost completely absent LR formation under severe N shortage [41]. These examples indicate that the availability of different nutrients can evoke distinct effects on root structure architecture that depend upon which nutrient is supplied and the concentration of the supplied nutrient. To deal with P-starvation, plants have developed several strategies to achieve localized P-sources.

**Fig. 3:**
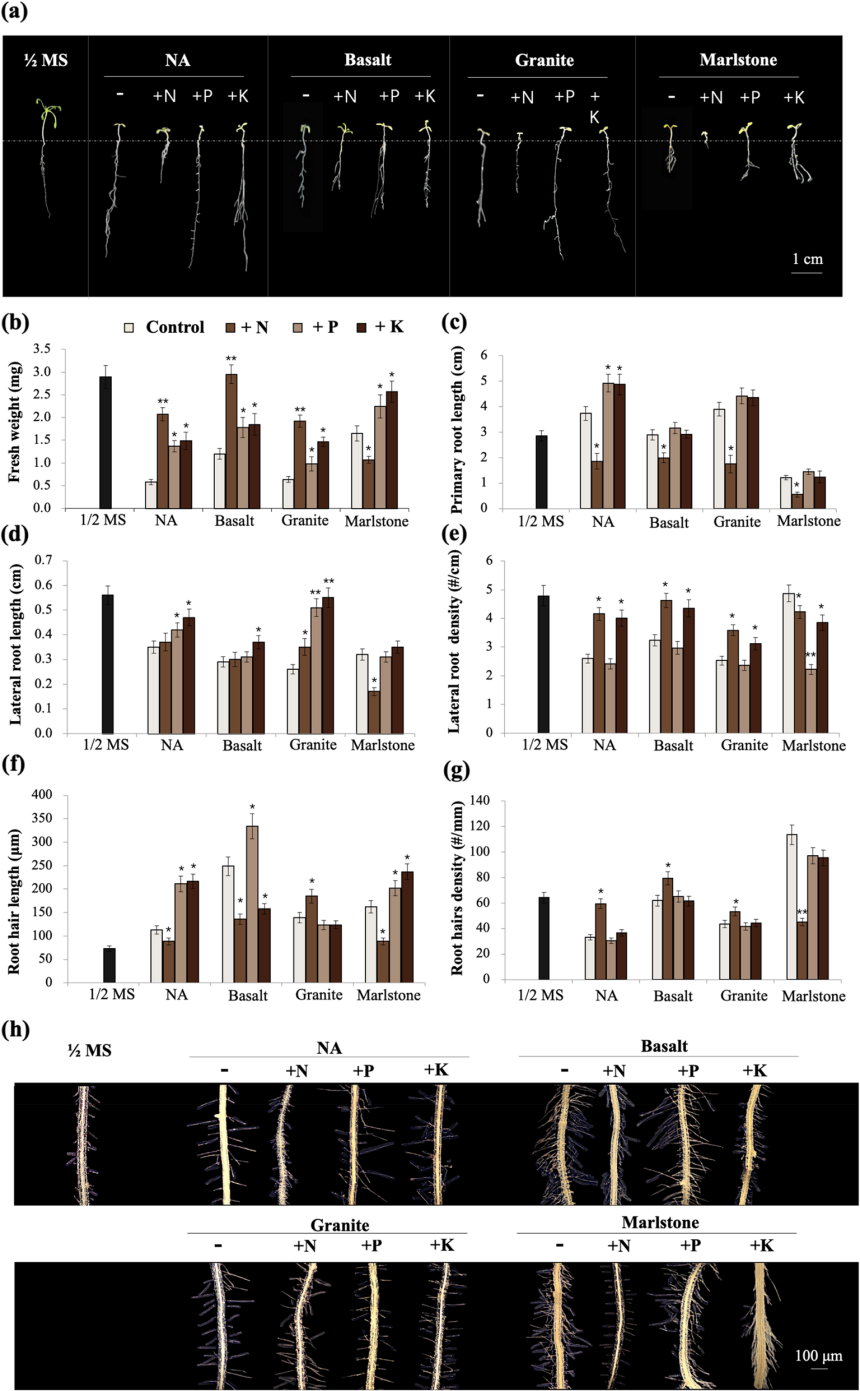
Effects of separate addition of N, P, and K elements to growth media on 2-week-old Arabidopsis seedling growth. **a** photographs of Arabidopsis seedlings with or without added nutrients; **b** fresh weight; **c** primary root length; **d** lateral root length; **e** lateral root density; **f** root hair length; **g** root hair density; **h** photographs showing root hairs. Data are presented as mean (*n* = 30) ± SD. * and ** indicate *p* < 0.05 and *p* < 0.01, respectively

In this work, we separately added N, P, and P back to the growth medium according to their respective original concentrations in the ½ MS formulation to study their individual effects on phenotypic recovery. In terms of fresh weight, the amendment of N, P, or K improved this growth parameter on all media, and of the three macronutrients, N proved most effective for plants grown on NA, basalt, and granite, whereas K was most effective for plants grown on marlstone, followed by P (Fig. 3a-b). However, there was one exception, i.e., the addition of N to the marlstone medium instead decreased the fresh weight of the seedlings (Fig. 3a-b).

Addition of N shortened the length of PR compared with plants growing on rock meal (Fig. 3c). Thus, N rescued the longer PR phenotype on NA, basalt, and granite, but exacerbated the shorter PR phenotype on marlstone, which may explain why fresh weight of plants on marlstone medium was further reduced by N addition (Fig. 3b). Unlike N, the addition of P and K to NA medium promoted the length of PR but only slightly increased this index when added to basalt, granite and marlstone (Fig. 3c). Taken together, our results suggest that although Arabidopsis seedlings grown on different rock media responded similarly to the addition of N, P, or K, their initial phenotypic characteristics should be considered in explaining their ultimate effects on plant growth.

As for the reduced average LR length of seedlings on NA, basalt, and granite, the addition of K, P, and N, in descending order, improved this parameter to varying degrees (Fig. 3d). But for the seedlings on marlstone medium, their LR length was further reduced by the addition of N (Fig. 3d). As for the density of LRs, the addition of N increased this parameter in seedlings grown on NA, basalt, and granite, we observed that the abnormal root growth was alleviated to some extent, which was similar to the “recovery effect” of the genetic complementation experiments. However, the LR density of seedlings on marlstone was suppressed by N addition, which was already evident in the changes in PR length and LR length. On NA, basalt, and granite, K increased LR density to a similar extent as N, while P showed little effect on this parameter (Fig. 3e). In contrast, P followed by K significantly decreased LR density on marlstone (Fig. 3e). Thus, based on the phenotypic changes in LR density, plants also responded differently to P and K on marlstone than on basalt and granite.

As for root hair length, the addition of N had a significant promoting effect only on seedlings growing on granite (Fig. 3f, h). However, on the other media, especially basalt and marlstone, root hairs became significantly shorter than those of seedlings growing only on rock flour (Fig. 3f, h). The addition of P promoted the length of root hairs on media other than granite, whereas the effect of K varied depending on the medium and was beneficial on NA and marlstone but inhibitory on basalt (Fig. 3f, h). Regarding the density of root hairs, the addition of N had various promoting effects on media other than marlstone and the effect of the addition of P and K on root hair density was not large (Fig. 3g, h).

### Auxin biosynthesis underlying N responses of seedlings on different rock meals

Auxin is differentially distributed in plant tissues and regulation of its metabolism and transport is important for plant developmental adaptations [42]. In Arabidopsis seedlings, auxin accumulates mainly at the root tip [43], particularly in the quiescent center (QC) and young columella cells [44, 45], a pattern required for the maintenance of a functional root meristem [46]. In this work, using a *DR5::GUS* marker line that reports auxin activity, we tracked the effects of mineral deficiency on the amount and location of auxin in the roots of Arabidopsis seedlings and attempted to determine why the addition of N increases seedling fresh weight on all media except marlstone.

**Fig. 4:**
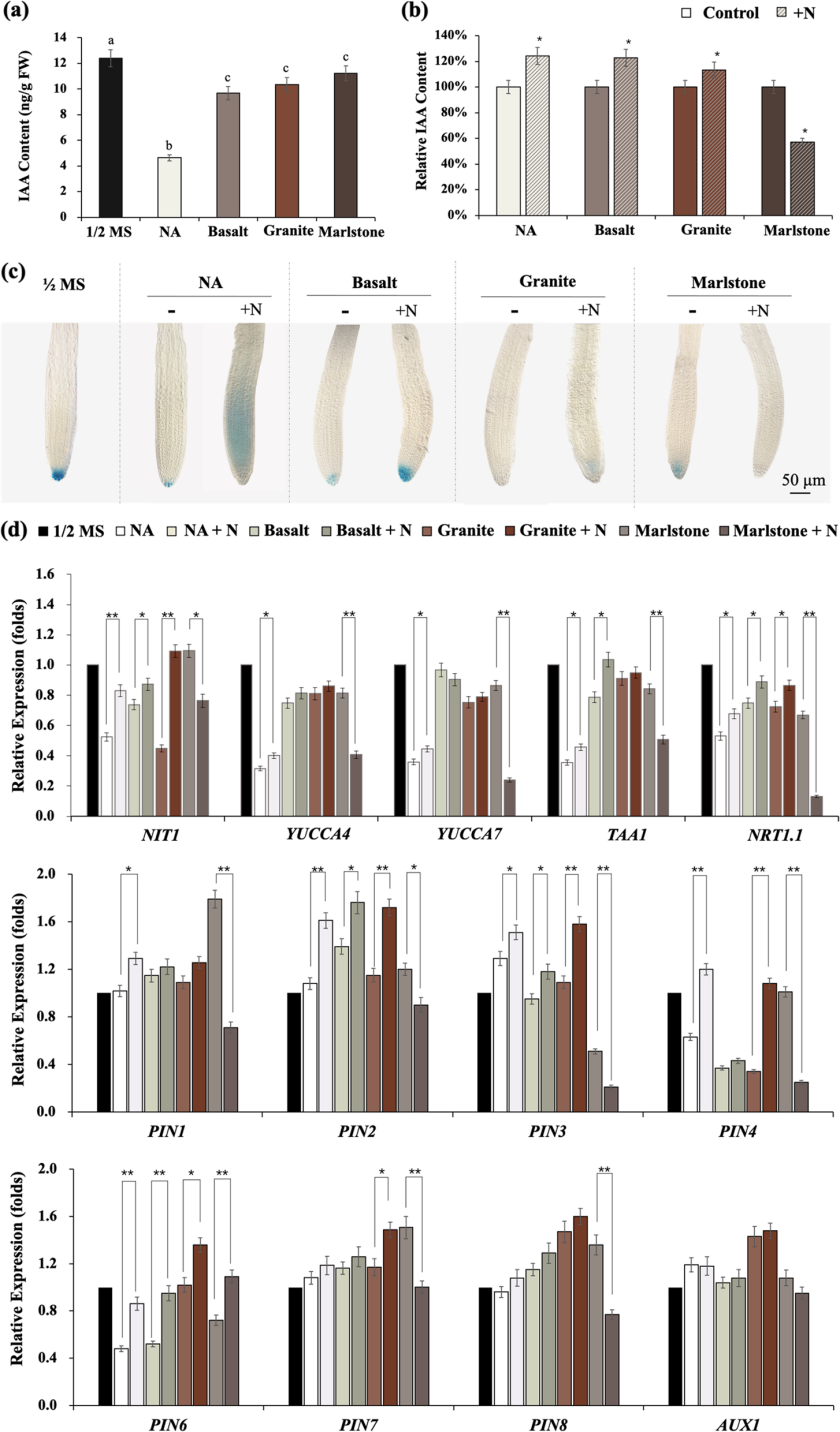
Effect of addition of N element to growth media on auxin biosynthesis of 2-week-old Arabidopsis seedlings. **a** Endogenous auxin level of *Arabidopsis* seedlings; **b** Distribution of auxin in root tips as shown in *DR5::GUS* lines; **c** The effect of N on the expression of genes involved in auxin biosynthesis as quantified by qRT-PCR. Data are expressed as mean (*n* = 3) ± SD. * and ** denote *p* < 0.05 and *p* < 0.01, respectively

First, we measured the auxin concentration of 14-day-old Arabidopsis seedlings on different media and found that the auxin concentration of seedlings grown on NA medium was only 37% of that of seedlings grown on ½ MS medium, whereas the auxin concentration of the other three seedlings ranged from 78 to 91% (Fig. 4a). After the addition of micronutrients and organics to the medium, the auxin content of plants growing on the four types of media was slightly, but not significantly, increased (Fig. S4). N significantly increased the auxin content of total seedlings on all rock meals except the marlstone medium (Fig. 4b). In addition, increased auxin content was observed with P addition on basalt and granite media (Fig. S5). We then analyzed the GUS staining patterns in the roots of *DR5::GUS* seedlings before and after N administration. It is worth noting that the intensity of GUS staining in the root tips of the seedlings was not consistent with the auxin levels measured in the whole seedlings (Fig. 4a, c), suggesting that in addition to moderately reduced auxin biosynthesis, auxin transport and/or local accumulation were likely severely impaired under nutrient deficiency.

However, consistent with the moderate increase in auxin content measured before and after the addition of N (Fig. 4b), diffuse and enhanced GUS staining was observed in the apical meristematic region of PR roots on NA media and stronger staining around the QC of roots on basalt and granite media (Fig. 4c). In contrast, N treatment essentially abolished the initial auxin signal on marlstone media (Fig. 4c). Thus, N administration resulted in opposite changes in auxin accumulation in root tips of seedlings growing on different rock media, which in turn could also explain the different responses in terms of LR initiation and elongation of seedlings (on NA, basalt, and granite, N resulted in increased density and length of LR, but on the marlstone medium, these indices decreased instead) since that auxin is the major endogenous regulator of LR organogenesis, and the LR developmental program at prebranching sites is usually triggered by auxin accumulation in the primary root [47]. To obtain a more complete picture of auxin regulation, the effect of N on the expression of genes involved in auxin biosynthesis including *TAA1*, *YUCCA 4*, *YUCCA* 7, and *NIT1*, was analyzed by quantitative RT-PCR. TAA1 and YUCCA-family members are the major players in auxin biosynthesis via the IPyA pathway [48]. While NIT1, which catalyzes indole-3-acetonitrile to IAA [49] highlights a more specific function for nitrilases to activate auxiliary pathways of IAA synthesis under certain circumstances [50]. Compared with gene expression on the ½ MS medium, expression of these genes was suppressed on the other four media, especially on the NA medium, which is consistent with the magnitude of the decrease in auxin levels measured at the whole seedling level (Fig. 4d). After the addition of N, the transcript abundance of these auxin biosynthetic genes was moderately upregulated on the NA, basalt, and granite media, except that it was drastically downregulated on the marlstone medium, consistent with the unusual decrease in auxin accumulation in the N-supplied root tips on marlstone (Fig. 4d).

To explain the disappearance of auxin accumulation in seedling root tips on N-supplied marlstone medium, we also analyzed the transcriptional changes of *NRT1.1*, a dual-affinity nitrate transporter and nitrate sensor [41, 51]. The results showed that the addition of N moderately increased the expression of *NRT1.1* in seedlings growing on the NA, basalt, and granite media, but drastically decreased its expression on the marlstone medium (Fig. 4d). Considering the function of NRT1.1 as a mediator of auxin uptake, its reduced transcriptional level could undermine auxin content and inhibit subsequent root branching. But why such modulation of NRT1.1-dependent auxin homeostasis occurred only on the marlstone medium and not on other media requires further investigation. We also investigated the changes in the expression of genes encoding the auxin influx carrier *AUX1/LAX* and the efflux carriers of the *PIN* family, which are the key rate-limiting components regulating auxin transport in root tips [52]. Shoot-derived IAA is mainly transported to columella cells by PIN1 and PIN4 [53, 54], and auxin synthesized by RAM is transported upward to the elongation zone by PIN2 [55]. PIN6 plays a role in the distribution of auxin during primary root growth as well as organogenesis and lateral root development [56, 57], and PIN7 has been reported to affect the total number of LRP initiated [43]. Interestingly, the addition of N to marlstone medium showed the opposite trend of change in gene expression compared with the addition of N to other nutrient-poor media, except for AUX1 and PIN6 (Fig. 4d), suggesting that the addition of N to different rock media might differentially affect PAT-mediated auxin transport and thus primary root elongation and LR initiation.

### Conclusion

Plants have evolved adaptive responses that allow them to grow in soils that contain small amounts of one or more nutrients. These responses require complex physiological and developmental changes to enhance the ability of plants to take up and remobilize nutrients. Although rock dust has been proposed as a long-term fertilizer and soil amendment, most research has focused on field experiments whose methodological inconsistencies have led to limited, scattered, and sometimes contradictory results. In this work, we investigated the nutrient availability of three representative natural rock meals on the growth of a model plant system, focusing on changes in root system architecture. Our data show that despite the generally extremely low mineral availability, plants perceive their different mineral profiles as a combinatorial signal and employ adaptive strategies by allocating finite resources in a differential manner that is closely linked to endogenous, hormonally controlled mechanisms. Considering that the soil environment is diverse and dynamic, such that there is no single equilibrium state of soil solution, these results provide a basis for revealing the presumed complexity of hormone-nutrient interactions at the biochemical and molecular levels and underscore the importance of understanding plant mineral supply at the systems level for the development of lower-input plant ideotypes and agricultural systems in the era of ‘agrogeology’.

### Supplementary Information

**Additional file 1: Table S1.** Primer sequences used in this work for qRT-PCR analysis. **Table S2.** Chemical composition of three rock powders. **Fig. S1.** Close-up images of Arabidopsis seedlings growing on horizontally arranged culture media. **Fig. S2.** Size effects of the three rock powders on Arabidopsis growth after sieving with 125 and 150 μm nylon mesh. (a) Changes in fresh weight; (b) changes in primary root length; (c) changes in lateral root length; (d) changes in lateral root density. Data are shown as mean (*n* = 30) ± SD. **Fig. S3.** Dosage effects (40% and 80% wt) on *Arabidopsis thaliana* of three rock meals after sieving with 150 μm nylon mesh. (a) Changes in fresh weight; (b) changes in primary root length; (c) changes in lateral root length; (d) changes in lateral root density. Data are shown as mean (*n* = 30) ± SD. **Fig. S4.** Effects of adding microelements and organic nutrients back to growth media on endogenous auxin content in 2-week-old Arabidopsis seedlings. Data are shown as mean (*n* = 3) ± SD. **Fig. S5.** Effects of adding P and K elements to growth media on endogenous auxin content in 2-week-old Arabidopsis seedlings. Data are shown as mean (*n* = 3) ± SD.

## Data Availability

All data generated or analysed during this study are included in this published article and its supplementary information files.
