# Supplementary material for: Arabidopsis seedlings respond differentially to nutrient efficacy of three rock meals by regulating root architecture and endogenous auxin homeostasis
Source: BMC Plant Biol. 2023 Dec 1;23:609. doi: 10.1186/s12870-023-04612-1 (PMC10691044; doi:10.1186/s12870-023-04612-1)

**Arabidopsis seedlings respond differentially to nutrient efficacy of three rock meals by regulating root architecture and endogenous auxin homeostasis**

Tianjiao Zhang^1^, Sainan Zhang^1^, Shaohui Yang^1^, Jianchao Zhang^2,*^, Jiehua Wang^1,*^ and H. Henry Teng^2^

^1^School of Environmental Science and Engineering, Tianjin University, Weijin Rd. 92, Nankai District, Tianjin, China, 300072

^2^School of Earth System Science, Institute of Surface-Earth System Science, Tianjin University, Weijin Rd. 92, Nankai District, Tianjin, China, 300072

*Corresponding authors, E-mail: Jiehua Wang: jiehuawang@tju.edu.cn; Jianchao Zhang: jianchaozhang@tju.edu.cn

**Table S1** Primer sequences used in this work for qRT-PCR analysis.

| **Gene name** |  | **Primer Sequences (5′ to 3′)** |
| --- | --- | --- |
| *NRT1.1* | F | TTCTCGTGACAATCGTCG |
|  | R | TGGAATACTCGGCTCATCAT |
| *NIT1* | F | GGCGTTCATAACGAAGAAGGGCGTG |
|  | R | TTCCTTCTCTATGGCTCCCATTACC |
| *YUCCA4* | F | ATGGGCACTTGTAGAGAATCAGA |
|  | R | AGCTAAACAATCGGTTCTCTCG |
| *YUCCA7* | F | GTTGTGTCAACATCTCAAGTATGCT |
|  | R | AACTTCTTGGCGTTTCAGGTC |
| *TAA1* | F | GTGAAGTTGTGAAAGAGAGCGAT |
|  | R | CTTGCTTATCTAAAGGTCAATGCT |
| *EF1α* | F | TGGTGACGCTGGTATGGTTA |
|  | R | AACTTCTTGGCGTTTCAGGTC |
| *AUX1* | F | AACCACTCTTGGGTCTTTCTTGC |
|  | R | GCCGTCGTGTTCTTCGTTATCT |
| *PIN1* | F | CGGTGGGAACAACATAAGCA |
|  | R | GGTGATGCCGAATAAACTGGA |
| *PIN2* | F | ACGGTTACACTAATAGCTACGG |
|  | R | CTTTCTTCGCCGTCTTCATAAC |
| *PIN3* | F | TCTTTGATTAGGTTCGGGTAACTC |
|  | R | GCTCATGTGAAACTGGAACAAG |
| *PIN4* | F | CAACGCCGTTAAATATGGA |
|  | R | AGACCCCATTTTATTCAGCC |
| *PIN6* | F | GGAGATTACACTCAAACCCTCA |
|  | R | CATCGGTTTCAGTTTCTGTACG |
| *PIN7* | F | CCGGAGTTTTCTACCGGTAATA |
|  | R | ACATTCGAATCTCTTTTGCACC |
| *PIN8* | F | CAAAGCTTGATTTGGTACACCA |
|  | R | ATTCCGATCAATGTTGCGTATG |

**Table S2** Chemical composition of three rock powders.

| **Basalt** | | |  | | **Granite** | | **Marlstone** | | | |  |
| --- | --- | --- | --- | --- | --- | --- | --- | --- | --- | --- | --- |
| SiO_2_ | | 4.43×10^5^ ppm |  | SiO_2_ | | 6.38×10^5^ ppm | |  | CaO | 2.60×10^5^ ppm | |
| Al_2_O_3_ | 1.62×10^5^ ppm |  | Al_2_O_3_ | | 1.18×10^5^ ppm | |  | SiO_2_ | 2.59×10^5^ ppm | |  |
| Fe_2_O_3_ | 9.02×10^4^ ppm |  | K_2_O | | 4.81×10^4^ ppm | |  | MgO | 1.07×10^5^ ppm | |  |
| CaO | 7.70×10^4^ ppm |  | Na_2_O | | 3.91×10^4^ ppm | |  | Al_2_O_3_ | 7.83×10^4^ ppm | |  |
| MgO | 5.71×10^4^ ppm |  | CaO | | 0.58×10^4^ ppm | |  | Fe_2_O_3_ | 4.78×10^4^ ppm | |  |
| Na_2_O | 3.62×10^4^ ppm |  | Fe_2_O_3_ | | 0.54×10^4^ ppm | |  | K_2_O | 3.95×10^4^ ppm | |  |
| K_2_O | 1.76×10^4^ ppm |  | MgO | | 0.18×10^4^ ppm | |  | TiO_2_ | 0.68×10^4^ ppm | |  |
| TiO_2_ | 1.38×10^4^ ppm |  | TiO_2_ | | 0.11×10^4^ ppm | |  | Na_2_O | 0.39×10^4^ ppm | |  |
| P_2_O_5_ | 0.66×10^4^ ppm |  | BaO | | 480 ppm | |  | MnO | 0.16×10^4^ ppm | |  |
| MnO | 0.14×10^4^ ppm |  | MnO | | 406 ppm | |  | P_2_O_5_ | 0.10×10^4^ ppm | |  |
| SrO | 851 ppm |  | Rb_2_O | | 174 ppm | |  | SO_3_ | 402 ppm | |  |
| BaO | 613 ppm |  | CeO_2_ | | 171 ppm | |  | Cl | 197 ppm | |  |
| V_2_O_5_ | 315 ppm |  | P_2_O_5_ | | 158 ppm | |  | BaO | 179 ppm | |  |
| ZrO_2_ | 274 ppm |  | Cl | | 123 ppm | |  | ZrO_2_ | 137 ppm | |  |
| Cr_2_O_3_ | 257 ppm |  | ZrO_2_ | | 73.7 ppm | |  | Cr_2_O_3_ | 121 ppm | |  |
| SO_3_ | 166 ppm |  | SrO | | 69.6 ppm | |  | SrO | 120 ppm | |  |
| ZnO | 109 ppm |  | SO_3_ | | 59.1 ppm | |  | ZnO | 85.7 ppm | |  |
| NiO | 104 ppm |  | CuO | | 32.3 ppm | |  | Rb_2_O | 80.0 ppm | |  |
| Cl | 63.7 ppm |  | ZnO | | 27.4 ppm | |  | CuO | 64.7 ppm | |  |
| CuO | 58.1 ppm |  | Ru | | 25.0 ppm | |  | NiO | 53.7 ppm | |  |
| Rb_2_O | 27.9 ppm |  | NiO | | 22.1 ppm | |  | As_2_O_3_ | 17.7 ppm | |  |
| Ga_2_O_3_ | 22.8 ppm |  | Ga_2_O_3_ | | 20.1 ppm | |  |  |  | |  |
| Y_2_O_3_ | 21.1 ppm |  | Y_2_O_3_ | | 17.2 ppm | |  |  |  | |  |
| Nb_2_O_5_ | 15.0 ppm |  | Nb_2_O_5_ | | 12.9 ppm | |  |  |  | |  |

**Supplementary Figure Legends**

**Fig. S1** Close-up images of Arabidopsis seedlings growing on horizontally arranged culture media.

**Fig. S2** Size effects of the three rock powders on Arabidopsis growth after sieving with 125 and 150 μm nylon mesh. (a) Changes in fresh weight; (b) changes in primary root length; (c) changes in lateral root length; (d) changes in lateral root density. Data are shown as mean (n = 30) ± SD.

**Fig. S3** Dosage effects (40% and 80% wt) on *Arabidopsis thaliana* of three rock meals after sieving with 150 μm nylon mesh. (a) Changes in fresh weight; (b) changes in primary root length; (c) changes in lateral root length; (d) changes in lateral root density. Data are shown as mean (n = 30) ± SD.

**Fig. S4** Effects of adding microelements and organic nutrients back to growth media on endogenous auxin content in 2-week-old Arabidopsis seedlings. Data are shown as mean (n = 3) ± SD.

**Fig. S5** Effects of adding P and K elements to growth media on endogenous auxin content in 2-week-old Arabidopsis seedlings. Data are shown as mean (n = 3) ± SD.

**Fig. S1**

**
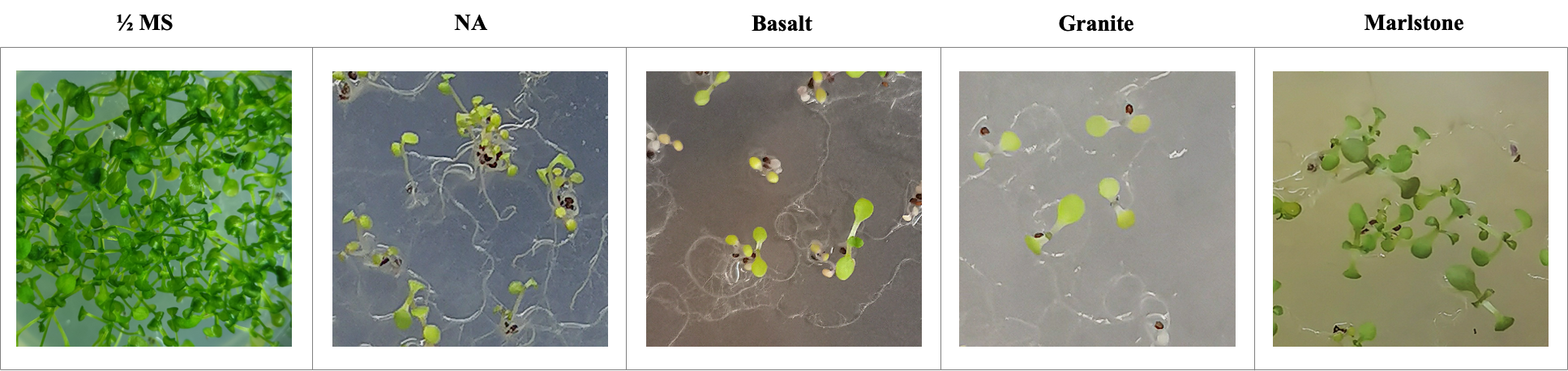
**

**Fig. S2**


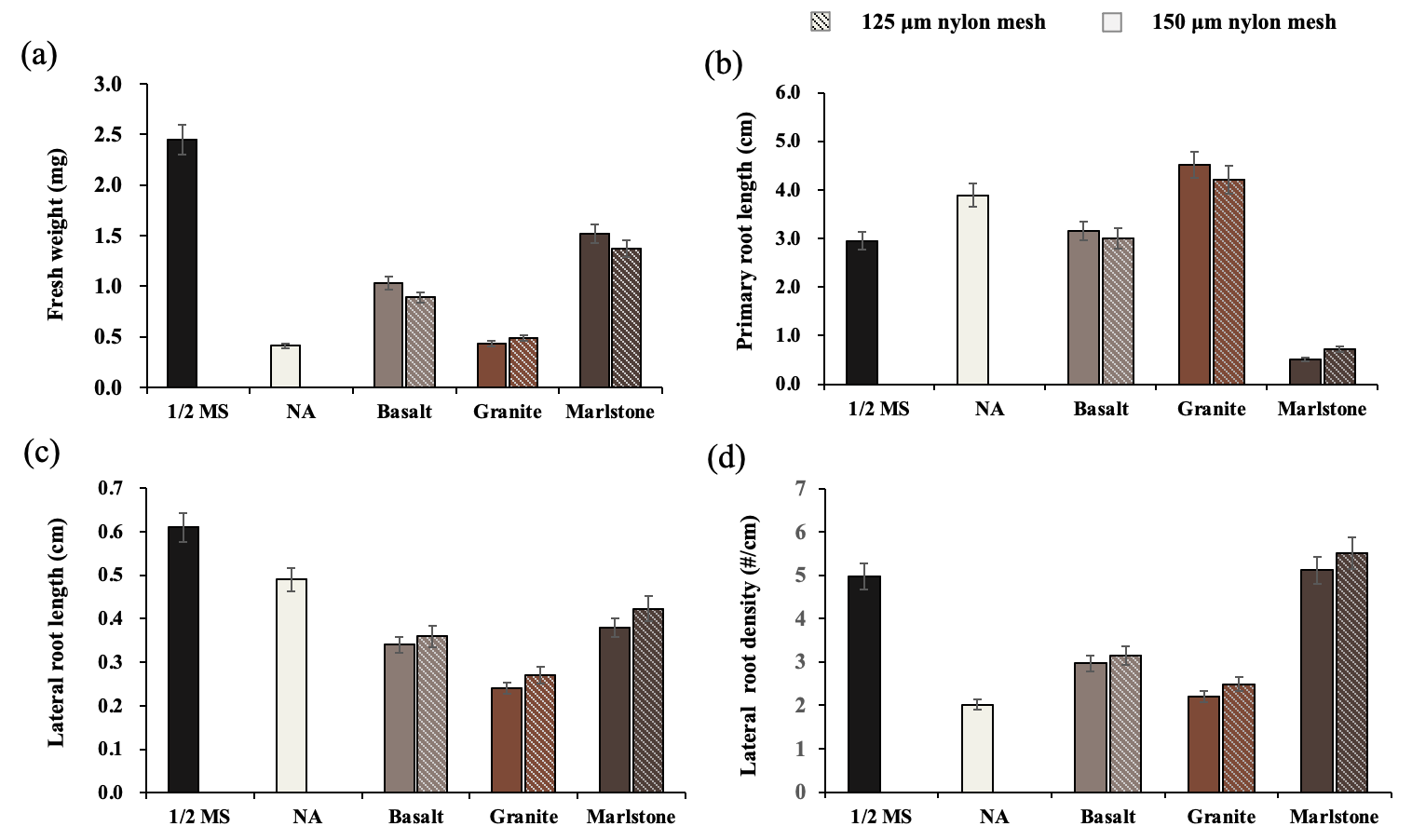


**Fig. S3**


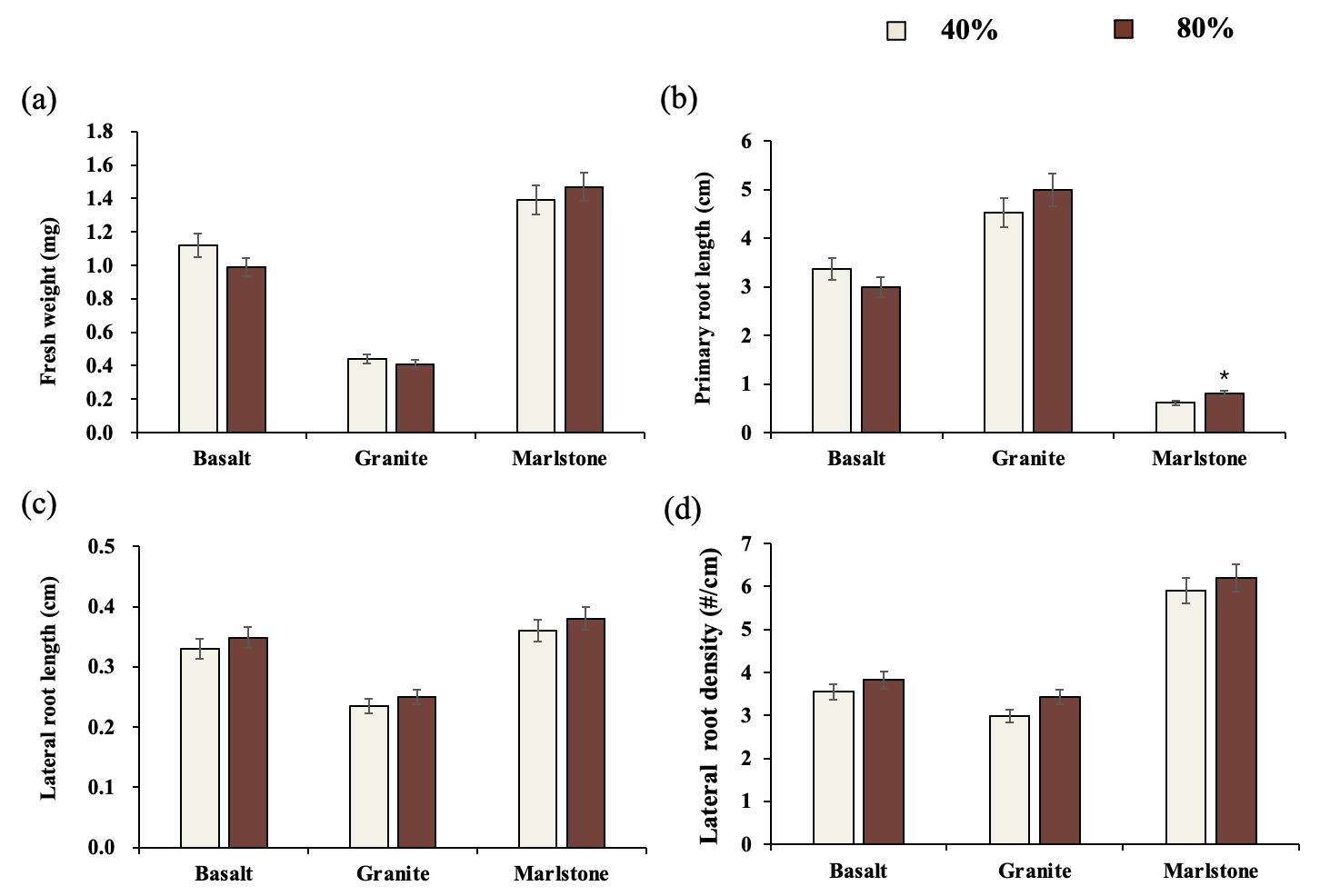


**Fig. S4**


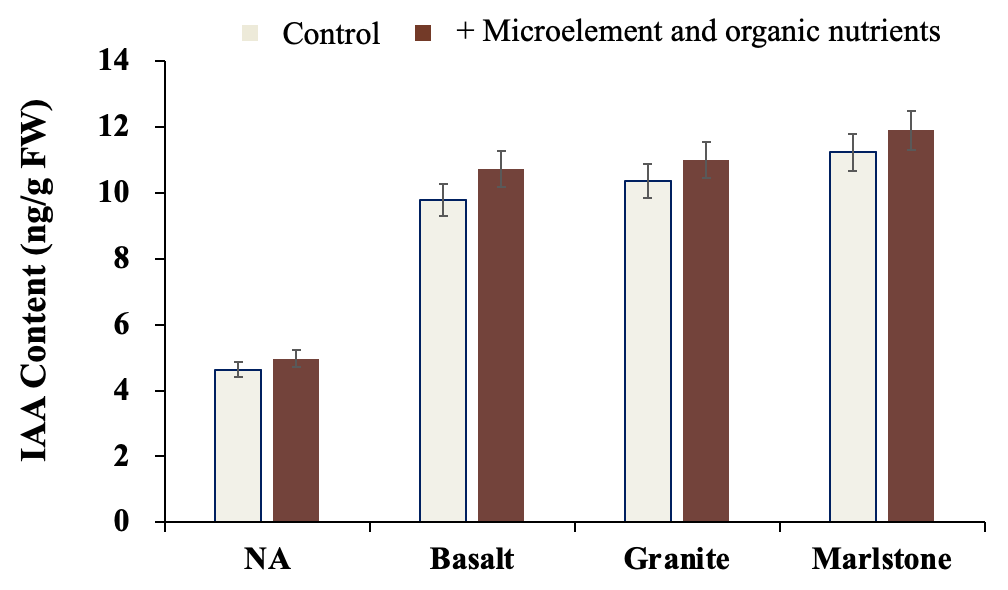


**Fig. S5**


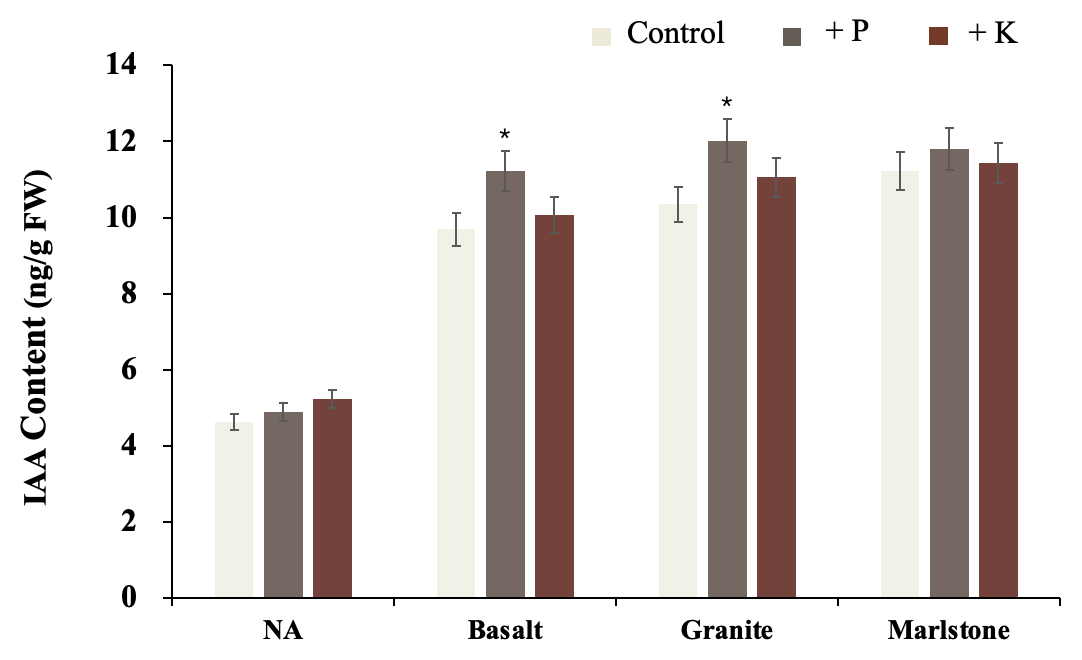

Supplement: Supplementary file 1 — Additional file 1: Table S1. Primer sequences used in this work for qRT-PCR analysis. Table S2. Chemical composition of three rock powders. Fig. S1. Close-up images of Arabidopsis seedlings growing on horizontally arranged culture media. Fig. S2. Size effects of the three rock powders on Arabidopsis growth after sieving with 125 and 150 μm nylon mesh. (a) Changes in fresh weight; (b) changes in primary root length; (c) changes in lateral root length; (d) changes in lateral root density. Data are shown as mean (n = 30) ± SD. Fig. S3. Dosage effects (40% and 80% wt) on Arabidopsis thaliana of three rock meals after sieving with 150 μm nylon mesh. (a) Changes in fresh weight; (b) changes in primary root length; (c) changes in lateral root length; (d) changes in lateral root density. Data are shown as mean (n = 30) ± SD. Fig. S4. Effects of adding microelements and organic nutrients back to growth media on endogenous auxin content in 2-week-old Arabidopsis seedlings. Data are shown as mean (n = 3) ± SD. Fig. S5. Effects of adding P and K elements to growth media on endogenous auxin content in 2-week-old Arabidopsis seedlings. Data are shown as mean (n = 3) ± SD. [file 12870_2023_4612_MOESM1_ESM.docx]
